# Remodeling of Tumor Stroma and Response to Therapy

**DOI:** 10.3390/cancers4020340

**Published:** 2012-03-27

**Authors:** Anna Johansson, Ruth Ganss

**Affiliations:** Western Australian Institute for Medical Research, Centre for Medical Research, University of Western Australia, Perth 6000, Australia; E-Mail: ajohansson@waimr.uwa.edu.au

**Keywords:** tumor stroma, tumor microenvironment, angiogenesis, immunotherapy

## Abstract

Solid tumors are intrinsically resistant to therapy. Cancer progression occurs when tumor cells orchestrate responses from diverse stromal cell types such as blood vessels and their support cells, inflammatory cells, and fibroblasts; these cells collectively form the tumor microenvironment and provide direct support for tumor growth, but also evasion from cytotoxic, immune and radiation therapies. An indirect result of abnormal and leaky blood vessels in solid tumors is high interstitial fluid pressure, which reduces drug penetration, but also creates a hypoxic environment that further augments tumor cell growth and metastatic spread. Importantly however, studies during the last decade have shown that the tumor stroma, including the vasculature, can be modulated, or re-educated, to allow better delivery of chemotherapeutic drugs or enhance the efficiency of active immune therapy. Such remodeling of the tumor stroma using genetic, pharmacological and other therapeutic approaches not only enhances selective access into tumors but also reduces toxic side effects. This review focuses on recent novel concepts to modulate tumor stroma and thus locally increase therapeutic efficacy.

## 1. Tumor Stroma: The Players

Tumor stromal cells are crucial for cancer initiation and progression. It has been highlighted in the last decade that tumor stroma is heterogeneous, highly dynamic and often tumor-type specific [1]. In general, the tumor microenvironment is composed of blood vessels, vascular support cells such as pericytes and smooth muscle cells, fibroblasts and various cells of the immune system. These cells either pre-exist in the tumor-originating organ or, alternatively, are recruited from the bone marrow and educated in the tumor environment [2]. One of the best studied entities of the tumor stroma is endothelial cells (EC) which form newly growing blood vessels. These vessels are supported by pericytes, a cell population of mesenchymal origin, which line ECs and supply paracrine survival factors. Fibroblasts and more specifically cancer associated fibroblasts (CAFs) comprise a large part of the tumor stroma and provide its structural framework by synthesis of extracellular matrix proteins. They also play an active role in shaping the tumor environment by secreting a wealth of growth factors [3]. Equally important are inflammatory cells, such as macrophages (also known as tumor associated macrophages, TAM), neutrophils and mast cells, which infiltrate solid tumors and create an immune suppressive environment which fosters tumor growth [4,5,6], affects clinical outcome [7,8] and/or therapeutic success [9]. Thus, growing tumors shape and educate surrounding stroma to cater for their needs which ultimately induces angiogenesis, immune evasion, tumor progression and metastases.

## 2. Tumor Blood Vessels: Kill or Not To Kill

Tumor angiogenesis, the process of creating new blood vessels through proliferation and migration of pre-existing ECs is induced by a dominance of pro-angiogenic over anti-angiogenic factors [10]. The importance of tumor angiogenesis was first recognized by Judah Folkman [11] who paved the way for a new concept which aimed at destroying tumor vessels to restrict oxygen and nutrient supplies to tumors. One of the major current targets for anti-angiogenic therapy is vascular endothelial growth factor (VEGF) which is highly expressed in the majority of tumors [12,13]. VEGF was initially discovered as vascular permeability factor (VPF) based on its ability to induce vessel leakiness [14] and later shown to be a secreted molecule that induces angiogenesis [15]. Vascular leakiness is a hallmark of all tumors. Paradoxically, however, leakiness for plasma molecules does not necessarily increase drug access. Instead, tumor vessels are unable to sustain an adequate blood flow. Reduced blood flow and perfusion whilst in an environment of increased metabolic demand from rapidly proliferating tumor cells creates tumor hypoxia and increased interstitial fluid pressure (IFP) [16,17,18]. High IFP in turn acts as barrier for effective drug delivery; it may also prevent infiltration of immune effector cells into the tumor parenchyma, an area which remains little studied to date [19,20]. In 2004, bevacizumab (Avastin^®^, Genentech/Roche), an anti-VEGF antibody, was the first FDA-approved angiogenesis inhibitor. Whilst disappointing as a single agent, it conferred significant survival benefits in patients with metastatic colorectal cancer when combined with chemotherapy [21]. However, not all patients respond to anti-VEGF therapy, responses are usually partial and ultimately, followed by relapse. Furthermore, recent studies in preclinical cancer models using a variety of VEGF-targeting strategies demonstrate a pattern of pro-invasive adaptation of anti-angiogenic therapy. For instance, treatment of tumor-bearing mice with VEGFR2-blocking antibodies, sunitinib (Sutent, Pfizer, a multi-targeted receptor tyrosine kinase inhibitor) or genetic deletion of VEGFA shows anti-tumor effects but also triggers local invasiveness and metastasis [22]. In a similar study, treatment with receptor tyrosine kinase inhibitors reduces growth of pre-established tumors, but increases metastatic tumor spreading when applied short-term or even before tumor cell inoculation. This indicates a high level of complexity with multi-targeted, anti-angiogenic drugs which is also dependent on scheduling and dosing [23]. These alarming findings imply that anti-angiogenic treatment strategies create new opportunities for tumor cells to adapt to altered environmental conditions with ensuing higher aggressiveness. In this context, several compensatory mechanisms have been postulated such as up-regulation of alternative angiogenic factors, recruitment of pro-angiogenic inflammatory cells into the tumor stroma, vessel stabilization through increased pericyte coverage and enhanced invasiveness of tumor cells into surrounding tissue [24]. It is imperative to further elucidate these resistance mechanisms and to develop combination therapies which may target other stromal compartments together with ECs for more durable effects. It is also worthwhile re-assessing the value of vascular destruction.

## 3. The Concept of Vascular Normalization

High IFP and hypoxia are certainly counterproductive for drug delivery, most standard-of-care cytotoxic- or radiation-therapies and potentially immunotherapy. Therefore, it is questionable whether enhancing hypoxia by killing tumor vessels will provide the best therapeutic outcome. Interestingly, an alternative concept to vascular destruction, namely “normalization” of tumor vessels has been proposed [18]. Vessel normalization in the context of pharmacological targeting of DNA topoisomerase II was described 40 years ago, at a time when Judah Folkman formulated his anti-angiogenesis hypothesis [25]. The concept of vessel normalization re-gained momentum when Jain and colleagues recognized that VEGF blockade can transiently reduce vascular permeability and vessel diameters in preclinical tumor models [26,27,28]. Subsequent studies demonstrated that vessel normalization reduces IFP and thus improves drug penetration into tumors [29]. For instance, in gliomas, a tumor type highly dependent on VEGF signaling, VEGF blockade improves tumor oxygenation and efficacy of radiation therapy [28,30]. This interesting concept also offers a plausible explanation for why anti-VEGF therapy is more effective when combined with chemotherapy. However, vessel normalization is not always observed after VEGF blockade and will require further validation to fully explore its translational potential [31].

Interestingly, independent of anti-VEGF therapy, “reversal” of angiogenesis has been observed in the context of immunotherapy [32]. So far, translation of immunotherapy into the clinic lags far behind other anti-cancer approaches [33]. Over the last decades numerous tumor antigens have been identified and strategies for effector cell activation have been optimized. Nevertheless, solid tumors and their immune suppressive environment still represent an obstacle for lymphocyte penetration and function which is reflected in modest clinical success [33,34]. More recently, we and others have reported that vessel activation which leads to expression of adhesion molecules on ECs dramatically increases lymphocyte access to solid tumors [32,35,36]. Interestingly, in human ovarian cancer, absence of tumor infiltrating lymphocytes and poor prognosis correlates with over expression of the endothelin B receptor. Blockade of the receptor leads to an up-regulation of intercellular adhesion molecule-1 (ICAM-1) and increased T cell homing into tumors [37]. In a model of pancreatic endocrine cancer, we have shown that local inflammation in tumors, e.g., induced by irradiation, induces endothelial activation and anti-tumor effector cell access into solid tumors [32]. Based on our findings, we postulated that tumor vessels represent a barrier for T cell infiltration and effective immunotherapy. Intriguingly, we also observed that in the process of immune rejection, tumor vessels were remodeled into a homogeneous, normalized network of smaller vessels with a more regular diameter. The parallels to vessel normalization under VEGF blockade are striking. However, vessel normalization is currently a highly descriptive term and underlying mechanisms are only beginning to emerge.

## 4. What Is Tumor Vessel Normalization?

In recent years, many laboratories have observed and analyzed “normalized” vessels under therapy. Morphologically, vascular normalization has been defined as a more organized, homogeneous vascular network with smaller vessel diameters [20]. Normalization is not restricted to endothelial cells but involves the whole vascular bed. For instance, pericyte coverage is an important parameter for the assessment of vessel remodeling. Similarly, vascular junction proteins that mediate adhesion between ECs such as zona occludens-1 (ZO-1), vascular endothelium cadherin (VE-Cadherin), and claudins are often re-arranged around normalized vessels [38]. Basement membrane (BM) components such as laminin [38] and collagen IV [28] are also remodeled. Collectively, these vascular alterations correlate with reduced leakiness and increased vessel perfusion. Improved vessel functionality in turn reduces tumor hypoxia and IFP. Increasing tumor oxygenation through normalization is a double-edged sword since it can promote primary tumor growth and as such is only viable in combination with other anti-tumor therapies; interestingly, however, it also reduces metastatic spread [38,39,40]. Whilst more information becomes available in different tumor models and indeed from cancer patients, it remains to be seen whether mechanisms of remodeling are shared between models and tumor types and, importantly, which stromal cells influence vascular remodeling.

## 5. The Role of Stromal Cells in Regulating Vascular Normalization and Tumor Progression

### 5.1. Pericytes

**Figure 1:**
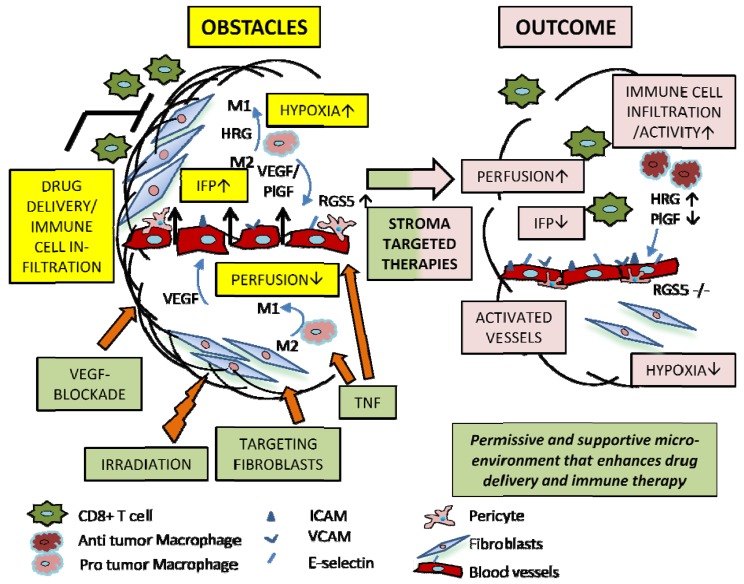
Targeting of tumor stroma. Various novel approaches targeting multiple components of the tumor stroma have been used to enhance vascular function and local perfusion and to create a permissive microenvironment for the improvement of drug delivery as well as immune therapy. For instance, pharmacological targeting of stroma derived VEGF [27] or genetic depletion of VEGF from macrophages [47], results in vascular normalization which enhances perfusion, drug delivery and chemotherapy [18]. Other key factors include PlGF produced by multiple cell types within the stroma including macrophages [48] and RGS5 expressed by pericytes [40]. Abbreviations: IFP, Interstitial Fluid Pressure; VEGF, Vascular Endothelial Growth Factor; TNF, Tumor Necrosis Factor; HRG, Histidine Rich glycoprotein; PlGF, Placental Growth Factor; RGS5, Regulator of G-protein Signaling 5; ICAM, Intercellular Adhesion Molecule; VCAM, Vascular Cell Adhesion Molecule.

Normalization of the vasculature can be influenced by different cells within the tumor microenvironment. In non-malignant tissue, pericytes and ECs are closely associated and stimulate each other via paracrine signaling. In tumors, however, pericyte coverage of ECs is abnormal showing weak and inconsistent attachments with large sleeves stretching out into the tumor parenchyma [41]. In addition, pericytes in tumors are immature and, at least in part, recruited from the bone marrow [42]. Pericytes are a dynamic cell population with high plasticity in tumors and unsurprisingly, their phenotype changes during vessel normalization. Recently, we provided the first evidence that pericytes also play a direct role in vascular remodeling. The molecule Regulator of G protein Signaling 5 (RGS5) is specifically expressed in platelet-derived growth factor receptor β-positive (PDGFRβ)^+^ immature pericytes during the angiogenic switch and further up-regulated in a highly angiogenic and hypoxic tumor environment [43,44]. Surprisingly, deletion of the RGS5 gene in pancreatic endocrine tumors induced vessel normalization (Figure 1). These vessels were covered by more mature pericytes which enhanced vessel functionality, improved tumor perfusion and oxygenation. As a net result, tumor growth was increased. However, vessel normalization opened tumors for infiltration of adoptively transferred, tumor-specific immune effector cells and subsequent tumor rejection [40]. These results are intriguing since they demonstrated for the first time that vessel normalization is sufficient to promote immune cell penetration into an otherwise inaccessible tumor environment. It still remains unclear whether immune cell access requires direct lymphocyte-EC interactions or is facilitated by reduced IFP. More recently, it has been shown that vessel normalization under VEGF-blockade also enhances active immunotherapy [45] which could indicate that both drug and immune cell penetration are increased via passive perfusion. Whilst effective immunotherapy also requires a supportive inflammatory environment in tumors [46], this finding has profound implications for the design of future combination therapies.

Low pericyte coverage correlates with poor clinical outcome in several different tumor types [49,50,51,52] but so far, the active involvement of pericytes in tumor progression remains unclear. Recently, specific depletion of neuron-glial antigen 2 (NG2) positive tumor pericytes was shown to suppress primary tumor growth but to enhance metastatic spread in mouse mammary tumors implying that pericytes may serve as negative regulators of metastasis [52]. Moreover, loss of pericytes resulted in marked increase in vascular leakage, hypoxia and epithelial to mesenchymal transition (EMT) of tumor cells which also drives invasiveness. In line with this, the frequency of lung metastasis was increased. Similarly, biopsies from human breast cancer showed that low pericyte coverage in combination with a high expression of c-Met (a marker for EMT) correlates with poor survival. These findings are of particular interest in the context of targeting pericytes together with ECs to potentiate anti-angiogenic therapy [53]. The rationale for this approach is that pericytes protect ECs during VEGF blockade [54], and may induce expression of survival signals and VEGF-A in ECs [55]. Results of combined targeting of EC and pericytes have so far been mixed with some laboratories reporting additive effects [53] whereas others have not found improvement over anti-VEGF therapy alone [56]. In light of Cooke *et al*.’s findings in breast cancer, targeting ECs alone may lead to transient vessel normalization, followed by delayed vessel loss, hypoxia and tumor invasiveness. Targeting pericytes alone or in combination with ECs may result in immediate vessel damage/leakiness, hypoxia and enhanced metastasis. Thus, pericytes play a pivotal role in controlling tumor perfusion and therapeutic outcome. Similar to ECs, there is increasing evidence which supports the rationale of direct pericyte targeting, however, not for destruction but to restore their maturity and EC support function.

### 5.2. Macrophages

Macrophages are innate immune cells which are found in the majority of solid tumors and by default promote angiogenesis and tumor growth [4]. An increasing number of subclasses are being identified indicating high plasticity within the population. The best characterized macrophages are TAMs, or M2 activated macrophages; their tumor promoting properties have been widely established in animal models [4,57,58] as well as in clinical settings [59]. TAMs drive tumor growth by secreting factors that stimulate breakdown of extracellular matrix and vessel growth, and inhibit anti-cancer immunity [60,61,62]. Circulating macrophages, TEMs (Tie2-expressing monocytes), are recruited from the bone marrow into growing tumors and often observed in close vicinity of blood vessels where they exert pro-angiogenic [63,64] and immune suppressive activities [65].

Importantly, macrophages have also been identified as regulators of vessel normalization. In an autochthonous breast cancer model, infiltrating myeloid cells express high levels of VEGF. Stockmann *et al*. demonstrated that myeloid-specific VEGF deletion normalizes tumor vessels [47] (Figure 1). These tumors harbor smaller, less tortuous vessels with increased pericyte coverage and show overall reduced hypoxia when compared to un-manipulated tumors. Similarly, Rolny *et al*. described vessel normalization in a histidine-rich glycoprotein (HRG) enriched tumor environment which is effected by macrophages. In HRG-rich tumors, TAMs are skewed from an M2 to a tumor-inhibiting M1 phenotype. Simultaneously, vessels are normalized resulting in reduced tumor hypoxia, increased delivery of cytotoxic drugs and decreased metastases (Figure 1). Moreover, TAM re-programming and vessel normalization substantially enhanced anti-tumor immunity by increasing the infiltration and activation of CD8^+^ T cells, dendritic cells and NK cells. Placental growth factor (PlGF), another angiogenic modulator, was identified as a key factor in this study. PlGF was down-regulated in re-programmed macrophages and specific deletion of PlGF in bone marrow-derived cells mimicked the anti-cancer effects of HRG [48].

In the context of immune-mediated tumor destruction, we had previously postulated that angiogenesis is a highly dynamic process that can be reversed in the “right” inflammatory context which also supports anti-tumor immunity [2,32]. It transpires now that macrophages, due to their high prevalence and plasticity in tumors, may well be a perfect target to create the “right” intratumoral inflammation. Impressively, re-education of macrophages in the tumor environment from a tumor-promoting (M2) to a tumor-inhibiting M1-like phenotype affects both vascular function and anti-tumor immunity [48]. Thus, HRG is a potential new anti-cancer agent which specifically polarizes macrophages to reduce PlGF secretion and increase pro-inflammatory cytokines. Interestingly, our own data suggest that low dose, local tumor necrosis factor alpha (TNFα) acts in a similar manner by re-educating macrophages to release inflammatory and angiogenic modulators which in turn remodel blood vessels and support anti-tumor immunity [66]. Taken together, these recent findings elucidating the role of macrophages in vessel normalization and cancer treatment suggest that targeting and re-polarizing macrophages is an attractive concept for future combination therapies.

### 5.3. Fibroblasts

Although CAFs have yet to be studied in the context of vascular normalization, their prevalence in tumor stroma, importance for tumor progression and capacity to modulate angiogenesis has long been recognized [3]. Early studies showed that co-injection of CAFs, but not normal fibroblasts, with prostate epithelial cells stimulates carcinogenesis [67]. CAFs are a rich source of growth factors such as VEGF and stromal-derived factor 1 (SDF1) [3], which promotes angiogenesis directly or by recruiting monocytes from the bone marrow [68,69]. Fibroblasts also contribute to the high IFP observed in tumors. Targeting PDGF receptors expressed on fibroblasts with a combination of Imatinib mesylate (Gleevec, Novartis) and chemotherapy has shown strong anti-tumor effects in several tumor models most likely by lowering IFP [70]. Another mechanism of action of Imatinib involves inhibition of angiogenesis through down-regulation of fibroblast growth factor 2 (FGF2) expressed by fibroblasts [71] or mast cells [8]. Recently, in mouse models of spontaneous gastrointestinal stromal tumors, Imatinib was also shown to activate CD8^+^ T cells and enhance concomitant immunotherapy [72].

Fibroblasts may directly or indirectly form a barrier for drug delivery into tumors. For instance, pancreatic ductal adenocarcinoma (PDA), a cancer with one of the poorest prognoses, is surrounded by a dense fibroblastic stroma and is inadequately vascularized and perfused. Standard-of-care chemotherapy is minimally effective in these patients. However, in a recent study, Olive *et al*. depleted stromal tissue in a mouse model of PDA using a hedgehog (Hh) signaling inhibitor (IPI-926) which specifically disrupts stromal signaling [73]. Interestingly, stromal destruction resulted in increased vascularity and drug penetration with significantly improved median survival. This study highlights the importance of fibroblasts as barriers to efficient drug delivery, as well as the need for adequate vascularisation to enable drug access into tumors.

To elucidate the nature of CAFs, gene signatures from normal and tumor-derived fibroblasts have been extensively studied [74,75,76]. For instance, in a mouse model of squamous skin carcinogenesis, fibroblasts are programmed at an early stage (dysplastic skin) to express a distinct set of pro-inflammatory genes. These inflammatory factors further amplify pro-tumorigenic inflammation, and drive angiogenesis and tumor growth in an nuclear factor kappa B (NFκB) dependent manner [77]. Blocking stromal NFκB signaling specifically abolished CAF-mediated, inflammatory effects and thus may be considered an adjuvant therapy with other anti-cancer strategies. Interestingly, Kraman *et al*. have shown that fibroblasts not only increase pro-tumorigenic inflammation but also actively suppress adoptive anti-tumor immunity [78]. Eliminating fibroblast activator protein-positive (FAP)^+^ fibroblasts from the tumor environment of Lewis lung carcinoma stimulates a tumor-specific immune response [78]. In a complex series of events, FAP^+^ cell depletion results in damage to the vasculature and hypoxic tumor necrosis which involves the cytokines interferon gamma (IFNγ) and TNFα. This in turn sets the stage for activation of tumor-specific T cells. Thus, depletion of fibroblasts from tumors represents a new concept for “priming” the tumor environment for immune-mediated rejection and can potentially be used as an adjuvant in combination with active immunotherapy. Interestingly, IFNγ has previously been shown to be essential for T cell-mediated tumor destruction through non-hematopoietic, stromal cells such as endothelial cells [79,80]. Recently, fibroblasts were also found to be crucial mediators of IFNγ’s anti-tumor immune effects through down-regulation of VEGF production and induction of angiostasis [81]. Therefore, CAFs are intimately involved in creating an inflammatory environment that supports tumor growth and inhibits anti-tumor immunity. Collectively, recent studies on CAFs show that fibroblasts are crucial components of the tumor microenvironment which represent a physical barrier for drug penetration, augment angiogenesis and sustain tumor-promoting inflammation. Indeed, they may represent important targets as stand-alone therapy, but more likely, as part of a combinatorial regimen involving chemotherapy or immunotherapy.

## 6. Conclusions

Cancer growth is crucially dependent on stromal interactions. Implicitly then, stromal targeting is critical for successful anti-cancer therapy. However, targeting tumor or stromal cells alone will have at best transient effects, as has been borne out empirically. Furthermore, promoting tumor hypoxia alone has not delivered sustained long term outcomes since it is prone to induce resistance, relapse and increased invasiveness. How then do we identify the best combination therapies? Therapeutic success will certainly be dependent on tumor type, size, location, stromal composition and accessibility. However, it is transpiring that stromal cells are dynamic in nature and can be re-educated to enhance therapeutic modalities such as cytotoxic, radiation, molecular targeted and immune therapies. We envision that best outcomes might be achieved in a tumor environment with low IFP and an inflammatory profile which supports anti-tumor immunity rather than tumor progression (Figure 1). This may be achieved by selectively depleting or re-programming crucial stromal components. Moreover, treatment modalities which act on multiple targets, simultaneously and sequentially, within the tumor environment may amplify beneficial effects, as well as prevent the emergence of resistant clones. Notably, successful anti-tumor approaches which focus on tumor or stromal targets seem to be more effective if they also revive adoptive tumor immunity [48,72,78]. The challenge ahead is to identify agents which can modulate cellular phenotypes and design stroma-specific targeting strategies.

## References

[1] Tlsty T.D., Coussens L.M. (2006). Tumor stroma and regulation of cancer development. Annu. Rev. Pathol..

[2] Ganss R. (2006). Tumor stroma fosters neovascularization by recruitment of progenitor cells into the tumor bed. J. Cell. Mol. Med..

[3] Kalluri R., Zeisberg M. (2006). Fibroblasts in cancer. Nat. Rev. Cancer.

[4] Mantovani A., Bottazzi B., Colotta F., Sozzani S., Ruco L. (1992). The origin and function of tumor-associated macrophages. Immunol. Today.

[5] Coussens L.M., Raymond W.W., Bergers G., Laig-Webster M., Behrendtsen O., Werb Z., Caughey G.H., Hanahan D. (1999). Inflammatory mast cells up-regulate angiogenesis during squamous epithelial carcinogenesis. Genes Dev..

[6] Nozawa H., Chiu C., Hanahan D. (2006). Infiltrating neutrophils mediate the initial angiogenic switch in a mouse model of multistage carcinogenesis. Proc. Natl. Acad. Sci. USA.

[7] Pages F., Galon J., Dieu-Nosjean M.C., Tartour E., Sautes-Fridman C., Fridman W.H. (2010). Immune infiltration in human tumors: A prognostic factor that should not be ignored. Oncogene.

[8] Johansson A., Rudolfsson S., Hammarsten P., Halin S., Pietras K., Jones J., Stattin P., Egevad L., Granfors T., Wikstrom P. (2010). Mast cells are novel independent prognostic markers in prostate cancer and represent a target for therapy. Am. J. Pathol..

[9] Shiao S.L., Ganesan A.P., Rugo H.S., Coussens L.M. (2011). Immune microenvironments in solid tumors: New targets for therapy. Genes Dev..

[10] Bergers G., Benjamin L.E. (2003). Tumorigenesis and the angiogenic switch. Nat. Rev. Cancer.

[11] Folkman J. (1971). Tumor angiogenesis: Therapeutic implications. N. Engl. J. Med..

[12] Zhang L., Yang N., Park J.W., Katsaros D., Fracchioli S., Cao G., O'Brien-Jenkins A., Randall T.C., Rubin S.C., Coukos G. (2003). Tumor-derived vascular endothelial growth factor up-regulates angiopoietin-2 in host endothelium and destabilizes host vasculature, supporting angiogenesis in ovarian cancer. Cancer Res..

[13] Tsuzuki Y., Fukumura D., Oosthuyse B., Koike C., Carmeliet P., Jain R.K. (2000). Vascular endothelial growth factor (VEGF) modulation by targeting hypoxia-inducible factor-1alpha → hypoxia response element → VEGF cascade differentially regulates vascular response and growth rate in tumors. Cancer Res..

[14] Senger D.R., Galli S.J., Dvorak A.M., Perruzzi C.A., Harvey V.S., Dvorak H.F. (1983). Tumor cells secrete a vascular permeability factor that promotes accumulation of ascites fluid. Science.

[15] Leung D.W., Cachianes G., Kuang W.J., Goeddel D.V., Ferrara N. (1989). Vascular endothelial growth factor is a secreted angiogenic mitogen. Science.

[16] Heldin C.H., Rubin K., Pietras K., Ostman A. (2004). High interstitial fluid pressure-An obstacle in cancer therapy. Nat. Rev. Cancer.

[17] Tredan O., Galmarini C.M., Patel K., Tannock I.F. (2007). Drug resistance and the solid tumor microenvironment. J. Natl. Cancer Inst..

[18] Jain R.K. (2005). Normalization of tumor vasculature: An emerging concept in antiangiogenic therapy. Science.

[19] Manzur M., Hamzah J., Ganss R. (2009). Modulation of G protein signaling normalizes tumor vessels. Cancer Res..

[20] Carmeliet P., Jain R.K. (2011). Principles and mechanisms of vessel normalization for cancer and other angiogenic diseases. Nat. Rev. Drug Discov..

[21] Hurwitz H., Fehrenbacher L., Novotny W., Cartwright T., Hainsworth J., Heim W., Berlin J., Baron A., Griffing S., Holmgren E. (2004). Bevacizumab plus irinotecan, fluorouracil, and leucovorin for metastatic colorectal cancer. N. Engl. J. Med..

[22] Paez-Ribes M., Allen E., Hudock J., Takeda T., Okuyama H., Vinals F., Inoue M., Bergers G., Hanahan D., Casanovas O. (2009). Antiangiogenic therapy elicits malignant progression of tumors to increased local invasion and distant metastasis. Cancer Cell.

[23] Ebos J.M., Lee C.R., Cruz-Munoz W., Bjarnason G.A., Christensen J.G., Kerbel R.S. (2009). Accelerated metastasis after short-term treatment with a potent inhibitor of tumor angiogenesis. Cancer Cell.

[24] Bergers G., Hanahan D. (2008). Modes of resistance to anti-angiogenic therapy. Nat. Rev. Cancer.

[25] Le Serve A.W., Hellmann K. (1972). Metastases and the normalization of tumour blood vessels by ICRF 159: A new type of drug action. Br. Med. J..

[26] Yuan F., Chen Y., Dellian M., Safabakhsh N., Ferrara N., Jain R.K. (1996). Time-dependent vascular regression and permeability changes in established human tumor xenografts induced by an anti-vascular endothelial growth factor/vascular permeability factor antibody. Proc. Natl. Acad. Sci. USA.

[27] Jain R.K. (2001). Normalizing tumor vasculature with anti-angiogenic therapy: A new paradigm for combination therapy. Nat. Med..

[28] Winkler F., Kozin S.V., Tong R.T., Chae S.S., Booth M.F., Garkavtsev I., Xu L., Hicklin D.J., Fukumura D., di Tomaso E. (2004). Kinetics of vascular normalization by VEGFR2 blockade governs brain tumor response to radiation: Role of oxygenation, angiopoietin-1, and matrix metalloproteinases. Cancer Cell.

[29] Tong R.T., Boucher Y., Kozin S.V., Winkler F., Hicklin D.J., Jain R.K. (2004). Vascular normalization by vascular endothelial growth factor receptor 2 blockade induces a pressure gradient across the vasculature and improves drug penetration in tumors. Cancer Res..

[30] Jain R.K., di Tomaso E., Duda D.G., Loeffler J.S., Sorensen A.G., Batchelor T.T. (2007). Angiogenesis in brain tumours. Nat. Rev. Neurosci..

[31] van der Veldt A.A., Lubberink M., Bahce I., Walraven M., de Boer M.P., Greuter H.N., Hendrikse N.H., Eriksson J., Windhorst A.D., Postmus P.E. (2012). Rapid decrease in delivery of chemotherapy to tumors after anti-VEGF therapy: Implications for scheduling of anti-angiogenic drugs. Cancer Cell.

[32] Ganss R., Ryschich E., Klar E., Arnold B., Hammerling G.J. (2002). Combination of T-cell therapy and trigger of inflammation induces remodeling of the vasculature and tumor eradication. Cancer Res..

[33] Sharma P., Wagner K., Wolchok J.D., Allison J.P. (2011). Novel cancer immunotherapy agents with survival benefit: Recent successes and next steps. Nat. Rev. Cancer.

[34] Ganss R., Arnold B., Hammerling G.J. (2004). Mini-review: Overcoming tumor-intrinsic resistance to immune effector function. Eur. J. Immunol..

[35] Garbi N., Arnold B., Gordon S., Hammerling G.J., Ganss R. (2004). CpG motifs as proinflammatory factors render autochthonous tumors permissive for infiltration and destruction. J. Immunol..

[36] Griffioen A.W. (2008). Anti-angiogenesis: Making the tumor vulnerable to the immune system. Cancer Immunol. Immunother..

[37] Buckanovich R.J., Facciabene A., Kim S., Benencia F., Sasaroli D., Balint K., Katsaros D., O'Brien-Jenkins A., Gimotty P.A., Coukos G. (2008). Endothelin B receptor mediates the endothelial barrier to T cell homing to tumors and disables immune therapy. Nat. Med..

[38] Mazzone M., Dettori D., Leite de Oliveira R., Loges S., Schmidt T., Jonckx B., Tian Y.M., Lanahan A.A., Pollard P., Ruiz de Almodovar C. (2009). Heterozygous deficiency of PHD2 restores tumor oxygenation and inhibits metastasis via endothelial normalization. Cell.

[39] de Bock K., Mazzone M., Carmeliet P. (2011). Antiangiogenic therapy, hypoxia, and metastasis: Risky liaisons, or not?. Nat. Rev. Clin. Oncol..

[40] Hamzah J., Jugold M., Kiessling F., Rigby P., Manzur M., Marti H.H., Rabie T., Kaden S., Grone H.J., Hammerling G.J. (2008). Vascular normalization in Rgs5-deficient tumours promotes immune destruction. Nature.

[41] Morikawa S., Baluk P., Kaidoh T., Haskell A., Jain R.K., McDonald D.M. (2002). Abnormalities in pericytes on blood vessels and endothelial sprouts in tumors. Am. J. Pathol..

[42] Song S., Ewald A.J., Stallcup W., Werb Z., Bergers G. (2005). PDGFRbeta^+^ perivascular progenitor cells in tumours regulate pericyte differentiation and vascular survival. Nat. Cell Biol..

[43] Berger M., Bergers G., Arnold B., Hammerling G.J., Ganss R. (2005). Regulator of G-protein signaling-5 induction in pericytes coincides with active vessel remodeling during neovascularization. Blood.

[44] Manzur M., Hamzah J., Ganss R. (2008). Modulation of the “blood-tumor” barrier improves immunotherapy. Cell Cycle.

[45] Shrimali R.K., Yu Z., Theoret M.R., Chinnasamy D., Restifo N.P., Rosenberg S.A. (2010). Antiangiogenic agents can increase lymphocyte infiltration into tumor and enhance the effectiveness of adoptive immunotherapy of cancer. Cancer Res..

[46] Hamzah J., Nelson D., Moldenhauer G., Arnold B., Hammerling G.J., Ganss R. (2008). Vascular targeting of anti-CD40 antibodies and IL-2 into autochthonous tumors enhances immunotherapy in mice. J. Clin. Invest..

[47] Stockmann C., Doedens A., Weidemann A., Zhang N., Takeda N., Greenberg J.I., Cheresh D.A., Johnson R.S. (2008). Deletion of vascular endothelial growth factor in myeloid cells accelerates tumorigenesis. Nature.

[48] Rolny C., Mazzone M., Tugues S., Laoui D., Johansson I., Coulon C., Squadrito M.L., Segura I., Li X., Knevels E. (2011). HRG inhibits tumor growth and metastasis by inducing macrophage polarization and vessel normalization through downregulation of PlGF. Cancer Cell.

[49] Yonenaga Y., Mori A., Onodera H., Yasuda S., Oe H., Fujimoto A., Tachibana T., Imamura M. (2005). Absence of smooth muscle actin-positive pericyte coverage of tumor vessels correlates with hematogenous metastasis and prognosis of colorectal cancer patients. Oncology.

[50] O'Keeffe M.B., Devlin A.H., Burns A.J., Gardiner T.A., Logan I.D., Hirst D.G., McKeown S.R. (2008). Investigation of pericytes, hypoxia, and vascularity in bladder tumors: Association with clinical outcomes. Oncol. Res..

[51] Stefansson I.M., Salvesen H.B., Akslen L.A. (2006). Vascular proliferation is important for clinical progress of endometrial cancer. Cancer Res..

[52] Cooke V.G., Lebleu V.S., Keskin D., Khan Z., O'Connell J.T., Teng Y., Duncan M.B., Xie L., Maeda G., Vong S. (2012). Pericyte Depletion results in hypoxia-associated epithelial-to-mesenchymal transition and metastasis mediated by Met signaling pathway. Cancer Cell.

[53] Bergers G., Song S., Meyer-Morse N., Bergsland E., Hanahan D. (2003). Benefits of targeting both pericytes and endothelial cells in the tumor vasculature with kinase inhibitors. J. Clin. Invest..

[54] Helfrich I., Scheffrahn I., Bartling S., Weis J., von Felbert V., Middleton M., Kato M., Ergun S., Schadendorf D. (2010). Resistance to antiangiogenic therapy is directed by vascular phenotype, vessel stabilization, and maturation in malignant melanoma. J. Exp. Med..

[55] Franco M., Roswall P., Cortez E., Hanahan D., Pietras K. (2011). Pericytes promote endothelial cell survival through induction of autocrine VEGF-A signaling and Bcl-w expression. Blood.

[56] Nisancioglu M.H., Betsholtz C., Genove G. (2010). The absence of pericytes does not increase the sensitivity of tumor vasculature to vascular endothelial growth factor-A blockade. Cancer Res..

[57] Lin E.Y., Nguyen A.V., Russell R.G., Pollard J.W. (2001). Colony-stimulating factor 1 promotes progression of mammary tumors to malignancy. J. Exp. Med..

[58] Pollard J.W. (2004). Tumour-educated macrophages promote tumour progression and metastasis. Nat. Rev. Cancer.

[59] Steidl C., Lee T., Shah S.P., Farinha P., Han G., Nayar T., Delaney A., Jones S.J., Iqbal J., Weisenburger D.D. (2010). Tumor-associated macrophages and survival in classic Hodgkin’s lymphoma. N. Engl. J. Med..

[60] Hagemann T., Biswas S.K., Lawrence T., Sica A., Lewis C.E. (2009). Regulation of macrophage function in tumors: The multifaceted role of NF-kappaB. Blood.

[61] DeNardo D.G., Andreu P., Coussens L.M. (2010). Interactions between lymphocytes and myeloid cells regulate pro- *versus* anti-tumor immunity. Cancer Metastasis Rev..

[62] Mantovani A., Sozzani S., Locati M., Allavena P., Sica A. (2002). Macrophage polarization: Tumor-associated macrophages as a paradigm for polarized M2 mononuclear phagocytes. Trends Immunol..

[63] de Palma M., Venneri M.A., Galli R., Sergi Sergi L., Politi L.S., Sampaolesi M., Naldini L. (2005). Tie2 identifies a hematopoietic lineage of proangiogenic monocytes required for tumor vessel formation and a mesenchymal population of pericyte progenitors. Cancer Cell.

[64] Mazzieri R., Pucci F., Moi D., Zonari E., Ranghetti A., Berti A., Politi L.S., Gentner B., Brown J.L., Naldini L. (2011). Targeting the ANG2/TIE2 axis inhibits tumor growth and metastasis by impairing angiogenesis and disabling rebounds of proangiogenic myeloid cells. Cancer Cell.

[65] Coffelt S.B., Chen Y.Y., Muthana M., Welford A.F., Tal A.O., Scholz A., Plate K.H., Reiss Y., Murdoch C., de Palma M. (2011). Angiopoietin 2 stimulates TIE2-expressing monocytes to suppress T cell activation and to promote regulatory T cell expansion. J. Immunol..

[66] Johansson A.C., Hamzah J., Payne C.J., Ganss R. (2012). Tumor targeted TNFα stabilizes tumor vessels and enhances active immunotherapy.

[67] Olumi A.F., Grossfeld G.D., Hayward S.W., Carroll P.R., Tlsty T.D., Cunha G.R. (1999). Carcinoma-associated fibroblasts direct tumor progression of initiated human prostatic epithelium. Cancer Res..

[68] Welford A.F., Biziato D., Coffelt S.B., Nucera S., Fisher M., Pucci F., di Serio C., Naldini L., de Palma M., Tozer G.M. (2011). TIE2-expressing macrophages limit the therapeutic efficacy of the vascular-disrupting agent combretastatin A4 phosphate in mice. J. Clin. Invest..

[69] Dineen S.P., Lynn K.D., Holloway S.E., Miller A.F., Sullivan J.P., Shames D.S., Beck A.W., Barnett C.C., Fleming J.B., Brekken R.A. (2008). Vascular endothelial growth factor receptor 2 mediates macrophage infiltration into orthotopic pancreatic tumors in mice. Cancer Res..

[70] Pietras K., Sjoblom T., Rubin K., Heldin C.H., Ostman A. (2003). PDGF receptors as cancer drug targets. Cancer Cell.

[71] Pietras K., Pahler J., Bergers G., Hanahan D. (2008). Functions of paracrine PDGF signaling in the proangiogenic tumor stroma revealed by pharmacological targeting. PLoS Med..

[72] Balachandran V.P., Cavnar M.J., Zeng S., Bamboat Z.M., Ocuin L.M., Obaid H., Sorenson E.C., Popow R., Ariyan C., Rossi F. (2011). Imatinib potentiates antitumor T cell responses in gastrointestinal stromal tumor through the inhibition of Ido. Nat. Med..

[73] Olive K.P., Jacobetz M.A., Davidson C.J., Gopinathan A., McIntyre D., Honess D., Madhu B., Goldgraben M.A., Caldwell M.E., Allard D. (2009). Inhibition of Hedgehog signaling enhances delivery of chemotherapy in a mouse model of pancreatic cancer. Science.

[74] Nakagawa H., Liyanarachchi S., Davuluri R.V., Auer H., Martin E.W., de la Chapelle A., Frankel W.L. (2004). Role of cancer-associated stromal fibroblasts in metastatic colon cancer to the liver and their expression profiles. Oncogene.

[75] Navab R., Strumpf D., Bandarchi B., Zhu C.Q., Pintilie M., Ramnarine V.R., Ibrahimov E., Radulovich N., Leung L., Barczyk M. (2011). Prognostic gene-expression signature of carcinoma-associated fibroblasts in non-small cell lung cancer. Proc. Natl. Acad. Sci. USA.

[76] Bauer M., Su G., Casper C., He R., Rehrauer W., Friedl A. (2010). Heterogeneity of gene expression in stromal fibroblasts of human breast carcinomas and normal breast. Oncogene.

[77] Erez N., Truitt M., Olson P., Arron S.T., Hanahan D. (2010). Cancer-associated fibroblasts are activated in incipient neoplasia to orchestrate tumor-promoting inflammation in an NF-kappaB-dependent manner. Cancer Cell.

[78] Kraman M., Bambrough P.J., Arnold J.N., Roberts E.W., Magiera L., Jones J.O., Gopinathan A., Tuveson D.A., Fearon D.T. (2010). Suppression of antitumor immunity by stromal cells expressing fibroblast activation protein-alpha. Science.

[79] Qin Z., Schwartzkopff J., Pradera F., Kammertoens T., Seliger B., Pircher H., Blankenstein T. (2003). A critical requirement of interferon gamma-mediated angiostasis for tumor rejection by CD8+ T cells. Cancer Res..

[80] Qin Z., Blankenstein T. (2000). CD4^+^ T cell-mediated tumor rejection involves inhibition of angiogenesis that is dependent on IFN gamma receptor expression by nonhematopoietic cells. Immunity.

[81] Lu Y., Yang W., Qin C., Zhang L., Deng J., Liu S., Qin Z. (2009). Responsiveness of stromal fibroblasts to IFN-gamma blocks tumor growth via angiostasis. J. Immunol..

